# Complete mitochondrial genome of the pearly razorfish *Xyrichtys novacula:* phylogenetic analysis of its placement within the Labridae family

**DOI:** 10.1080/23802359.2019.1711226

**Published:** 2020-01-16

**Authors:** Margarida Barcelo-Serra, Joan Pons, Tomeu Viver, Ramon Rosselló-Mora, Josep Alós

**Affiliations:** Institut Mediterrani d’Estudis Avançats, IMEDEA (UIB-CSIC) Illes Balears, Spain

**Keywords:** Labridae, mitochondrial genome, mitogenome, pearly razorfish, *Xyrichtys novacula*

## Abstract

In this paper, we report the complete mitochondrial genome (17,306 bp) of the pearly razorfish *Xyrichtys novacula* Linnaeus, 1758, a labrid that inhabits tropical and temperate Atlantic waters and the Mediterranean Sea. The circular double-stranded sequence contains the typical teleost gene order with 13 protein-coding genes, 22 tRNA, 2 rRNA, 1 control region, and 2 intergenic spacers between the rRNAs. Using the sequences of all protein-coding genes, we inferred the phylogeny for the Labriade family using 24 labrids and 3 outgroup species that placed *X. novacula* in a monophyletic group including species from the Pseudocheilines, Pseudolabrines, and Julidines.

The Labridae (wrasses) is an extensive family (82 genera and about 600 species) of teleost fishes found in coastal temperate and tropical marine waters worldwide (Nelson et al. 2016). In this paper, we studied the pearly razorfish (*Xyrichtys novacula,* novaculines), a labrid species exploited by fisheries in tropical and temperate coastal waters of the Eastern and Western Atlantic and the Mediterranean Sea (Alós et al. 2012, 2016). The phylogeny of the Labridae has been previously reported (Westneat and Alfaro 2005), but the pearly razorfish was not considered. Furthermore, important genetic differences have been found among Atlantic and Mediterranean populations, suggesting different cryptic species across *X. novacula* (Nirchio et al. 2019). The information we provide can be useful for future phylogenetic and taxonomic studies on the pearly razorfish and closely related taxa.

The specimen analyzed in this study was obtained from a Mediterranean wild population in the Palma Bay, Mallorca, Spain (39.439775 N, 2.732251E). The specimen has been deposited and registered at the Mediterranean Institute for Advanced Studies Collection (ID: IMEDEA108426). For sequencing purposes, intestinal tissue was excised using a sterile scalpel, stored in RNA later and frozen at −80 °C. The DNA extraction was performed as detailed in Urdiain et al. (2008). Paired-end libraries were constructed using Nextera DNA Flex Library Prep and sequenced (150 bp fragment length) by FISABIO (Valencia, Spain) using Illumina NextSeq. We used SolexaQA tool v3.1.4. (Cox et al. 2010) to trim the paired-end reads with a quality score below 20 and discarded fragments <50 bp in length. The trimmed reads were assembled with IDBA v1.1.1 assembler (Peng et al. 2012) using the “–pre-correction” option. The complete mitogenome contig sequence was annotated and validated using MITOS2 (Bernt et al. 2013) and tbl2asn and submitted to GeneBank (accession number MN794015).

The circular mitogenome is 17,306 bp in length with slight A–T bias 52.8%. It has 13 protein-coding genes (PCG, 12 in the plus strand and *nad6* in the minus), with start and stop codons matching the reported for other Labridae (Niu et al. 2018; Guo et al. 2019; Song et al. 2019) except for stop codon (TAG) in *nad1*, *nad2*, *nad3,* and *nad5*, and stop codon (TAA) in *atp6* and *cox3*. The genome contains 22 tRNA (14 in the plus strand and *trnS2, trnE, trnP, trnQ, trnA, trnN, trnC*, and *trnY* in the minus), 12S and 16S rRNA in the plus strand, a 921 bp control region, and two intergenic spacers between the ribosomal genes, which have been previously described in other teleosts (Zhuang et al. 2013).

We obtained mitochondrial PCG sequences for 23 labrids and three outgroups from GeneBank (accession numbers in Figure 1). We aligned each PCG using TranslatorX (Abascal et al. 2010) and used a concatenated sequence in iq-tree v.1.6.8 (Nguyen et al. 2015) to find the best partition scheme (-sp command), evolutionary models, tree topology, branch lengths, and fast bootstrap support (Figure 1). We did not include *nad5* and *nad6* for *P. flagellifer* and *P. eoethinus* due to suspected chimerism in the GeneBank sequences. The genus *Xyrichtys* was placed within a monophyletic group including species of the wrasses groups Pseudocheilines, Pseudolabrines, and Julidines, consistent with Westneat and Alfaro (2005). The gene rearrangements detected in the phylogeny by the software Trex (http://pacosy.informatik.uni-leipzig.de/185-0-TreeREx.html) are detailed in Figure 1.

**Figure:**
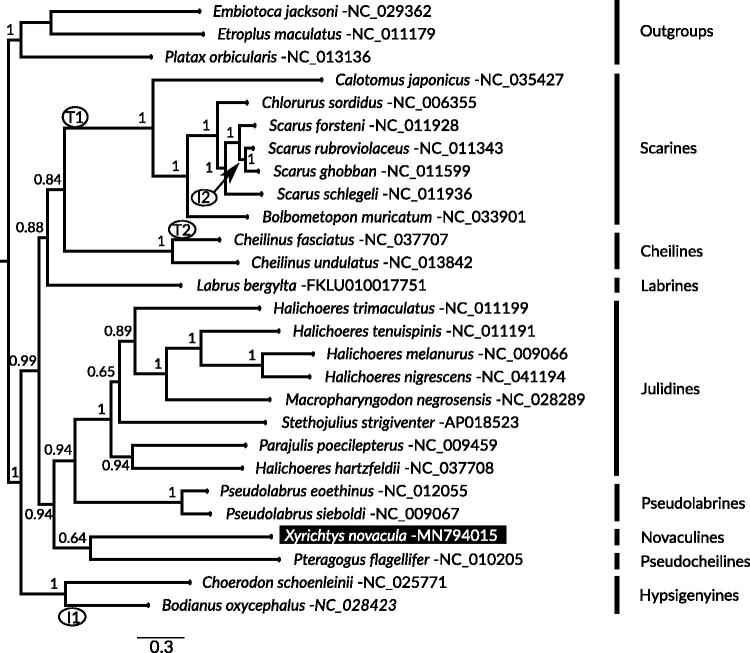
**Figure1.** Maximum likelihood phylogenetic tree highlighting the placement of *Xyrichtys novacula*. The bootstrap support values were obtained via 1000 pseudoreplicates. Gene rearrangements are circled and marked as I1 (inversion *trnQ*), I2 (inversion *trnP*), T1 (transposition *trnQ*, *trnM*), T2 (transposition *trnH*, *trnS1*). The best partition schemes and models were: 1st codon sites (GTR + I+G), 2nd codon positions (GTR + I+G), and 3rd codon nucleotides (GTR + I+G). The GeneBank accession number follows the name of each species.

## References

[CIT0001] Abascal F, Zardoya R, Telford MJ. 2010. TranslatorX: multiple alignment of nucleotide sequences guided by amino acid translations. Nucleic Acids Res. 38(suppl_2):W7–W13. 20435676 10.1093/nar/gkq291 PMC2896173

[CIT0002] Alós J, Cabanellas-Reboredo M, Lowerre-Barbieri S. 2012. Diel behaviour and habitat utilisation by the pearly razorfish during the spawning season. Mar Ecol Prog Ser. 460:207–220.

[CIT0003] Alós J, Palmer M, Rosselló R, Arlinghaus R. 2016. Fast and behavior-selective exploitation of a marine fish targeted by anglers. Sci Rep. 6(1):13. 27922022 10.1038/srep38093 PMC5138602

[CIT0004] Bernt M, Donath A, Jühling F, Externbrink F, Florentz C, Fritzsch G, Pütz J, Middendorf M, Stadler P F. 2013. MITOS: Improved de novo metazoan mitochondrial genome annotation. Mol Phylogenet Evol. 69(2):313–319. 22982435 10.1016/j.ympev.2012.08.023

[CIT0005] Cox MP, Peterson DA, Biggs PJ. 2010. SolexaQA: at-a-glance quality assessment of Illumina second generation sequencing data. BMC Bioinformatics. 11(1):485. 20875133 10.1186/1471-2105-11-485 PMC2956736

[CIT0006] Guo H-Y, Zhang N, Zhu K-C, Liu B-S, Guo L, Yang J-W, Zhang D-C. 2019. Characterization of the complete mitochondrial genome sequence of orangeline wrasse *Halichoeres hartzfeldii* (Bleeker, 1852) with the phylogenetic relationships within the Labridae species. Mitochondr DNA B. 4(2):3254–3255. 10.1080/23802359.2019.1669501 PMC7707247 33365943

[CIT0007] Nelson JS, Grande T, Wilson MVH. 2016. Fishes of the world, Fifth Edit. John Wiley & Sons, Inc., Hoboken, New Jersey.

[CIT0008] Nguyen L-T, Schmidt HA, von Haeseler A, Minh BQ. 2015. IQ-TREE: A fast and effective stochastic algorithm for estimating maximum-likelihood phylogenies. Mol Biol Evol. 32(1):268–274. 25371430 10.1093/molbev/msu300 PMC4271533

[CIT0009] Nirchio M, Gaviria J I, Siccha-Ramirez Z R, Oliveira C, Foresti F, Milana V, Rossi A R. 2019. Chromosomal polymorphism and molecular variability in the pearly razorfish *Xyrichtys novacula* (Labriformes, Labridae): taxonomic and biogeographic implications. Genetica. 147(1):47–56. 30673915 10.1007/s10709-019-00051-9

[CIT0010] Niu W, Kong L, Ma H, Gao Y. 2018. Characterization and phylogenetic analysis of the complete mitochondrial genome of *Bodianus oxycephalus* (Perciformes, Labridae). Conservation Genet Resour. 10(3):491–494.

[CIT0011] Peng Y, Leung HC, Yiu SM, Chin F. 2012. IDBA-UD: a de novo assembler for single-cell and metagenomics sequencing data with highly uneven depth. Bioinformatics. 28(11):1420–1428. 22495754 10.1093/bioinformatics/bts174

[CIT0012] Song H Y, Jung S-H, Jo S, Hwang H-J, Kim S-Y, An H S. 2019. Complete mitochondrial genome of *Stethojulis Strigiventer* (Labriformes, Labridae): mitogenome characterization and phylogenetic analysis. Mitochondr DNA B. 4(1):908–909.

[CIT0013] Urdiain M, López-López A, Gonzalo C, Busse H-J, Langer S, Kämpfer P, Rosselló-Móra R. 2008. Reclassification of *Rhodobium marinum* and *Rhodobium pfennigii* as Afifella marina gen. nov. comb. nov. and *Afifella pfennigii* comb. nov., a new genus of photoheterotrophic Alphaproteobacteria and emended descriptions of Rhodo. Syst Appl Microbiol. 31(5):339–351. 18774253 10.1016/j.syapm.2008.07.002

[CIT0014] Westneat MW, Alfaro ME. 2005. Phylogenetic relationships and evolutionary history of the reef fish family Labridae. Mol Phylogenet Evol. 36(2):370–390. 15955516 10.1016/j.ympev.2005.02.001

[CIT0015] Zhuang X, Qu M, Zhang X, Ding S. 2013. A comprehensive description and evolutionary analysis of 22 grouper (Perciformes, Epinephelidae) mitochondrial genomes with emphasis on two novel genome organizations. PLOS One. 8(8):e73561. 23951357 10.1371/journal.pone.0073561 PMC3739747

